# Editorial: Pine bark extract: nutrition and metabolism

**DOI:** 10.3389/fnut.2024.1481632

**Published:** 2024-09-13

**Authors:** Ying-Yu Cui, Maria Daglia, Hammad Ullah

**Affiliations:** ^1^Department of Cell Biology, Institute of Medical Genetics, State Key Laboratory of Cardiology, Tongji University School of Medicine, Shanghai, China; ^2^Department of Pharmacy, University of Napoli Federico II, Naples, Italy; ^3^International Research Center for Food Nutrition and Safety, Jiangsu University, Zhenjiang, China

**Keywords:** pine bark extracts, proanthocyanidins, metabolic kinetics, metabolic disease, nutritional therapy

Pine bark extracts (PBEs) were previously considered to be an inconvenient waste product in the timber industry but are now widely acknowledged as a rich source of natural polyphenols, mainly with potential nutritional, health, and medicinal properties (1). Proanthocyanidins are among the most abundant constituents in PBEs, at least including Pycnogenol (PYC) (2) from the bark of the European coastal or French maritime pine *Pinus pinaster*, Enzogenol^®^ from the bark of New Zealand pine *Pinus radiata* (Monterey or Insignis pine) (3) and PMBE from the bark of China pine *Pinus massoniana* (4–8). Owing to the presence of proanthocyanidins, these extracts possess range of health benefits including antioxidant, anti-inflammatory, cardioprotective, antimutagenic, antimetastatic, anticarcinogenic, and neuroprotective properties, directly and/or indirectly by interacting with other components and/or via intestinal microflora participating in their metabolism (9). Despite the fact that earlier studies have advanced our knowledge of PBE's role in food formulation and processing, as well as their use in pharmaceuticals or nutraceuticals (for instance, as antioxidants in the meat industry and as additives in the production of juice, wine, and noodles) (10), it is still difficult to uncover the cellular and molecular mechanisms of PBE functioning due to a variety of factors, including different growth environments, extraction techniques, cell lines, cell culture media, and animal models. In particular, its metabolic kinetics in living organisms remains unclear. Therefore, more research is required to comprehend the metabolic properties of PBE from different origins, with different growth environment, and for diverse applications.

This Research Topic aimed to explore the detailed chemical characterization of different types of PBE, the isolation and purification of the potentially active metabolites, and metabolic kinetic models for a better understanding of the nutritional and pharmacological potential of PBE. This Research Topic contributes to fill the gap in the knowledge about dietary bioactive compounds of PBE by presenting current and novel methodologies being used to obtain information about their presence, concentration, availability and activity in different food products, and their effects on different tissues and organs of human body, thereby deepening human understanding of the multi-component, multi-target, and multi-signal pathway mechanisms of PBE, and conceit R&D styles and methods as food ingredients and preservatives as well.

This Research Topic currently collects five articles including two review papers, two original research papers and one clinical trial. Sánchez-Moya et al. reported their findings that PBE and/or its phenolic constituents could function as protectors against gastrointestinal disturbances which lead to ulcerative colitis and Crohn's disease. Liu et al. demonstrated the anti-inflammatory ingredients and molecular mechanisms of pycnogenol^®^ via integrating network pharmacology and a series of verification experiments. Range of analysis suggested that pycnogenol^®^ could affect inflammation via multi-components, multi-targets, and muti-mechanisms. Weichmann and Rohdewald summarized randomized, placebo-controlled human clinical studies on the effects of French maritime pine bark extract (Pycnogenol^®^), giving a comprehensive overview of its potential benefits across various health domains, and providing a basis for further research on its applications in nutraceutical and pharmaceutical sectors. While reviewing the pharmacokinetics of French maritime pine bark extract (Pycnogenol^®^) in humans, Bayer and Högger concluded that low molecular weight constituents of the extract, such as catechin, caffeic acid, ferulic acid, and taxifolin are readily absorbed from the small intestine into systemic circulation, whereas, procyanidin oligomers and polymers are subjected to gut microbial degradation in the large intestine yielding small bioavailable metabolites such as 5-(3′,4′-dihydroxyphenyl)-γ-valerolactone. After intake of Pycnogenol^®^, constituents and metabolites have also been detected in blood cells, synovial fluid and saliva indicating a substantial distribution in compartments other than serum. In studies simultaneously investigating concentrations in different specimen, a preferential distribution of individual compounds [such as ferulic acid and 5-(3′,4′-dihydroxyphenyl)-γ-valerolactone] has been observed into synovial fluid. Renal excretion is reported as the main route of elimination of constituents and metabolites of the French pine bark extract. Briefly, the extensive body of knowledge pertaining to the pharmacokinetics of Pycnogenol^®^ components and their metabolites provides a rational basis for their effects, observed in preclinical and clinical studies. Weyns et al. explored a potential prebiotic effect of the polyphenol-rich French Maritime PBE compared to methylphenidate (MPH) and a placebo in pediatric Attention-Deficit Hyperactivity Disorder (ADHD) patients. Although their small sample size (placebo: *n* = 10; PBE: *n* = 13; MPH: *n* = 14) did not allow to observe clear prebiotic effects in the patients treated with PBE, subtle noticeable changes and some observed limited compositional changes throw light upon pediatric ADHD treatment via gut microbiota regulation through PBE and MPH interventions.

In conclusion, this Research Topic offered multidisciplinary investigations on the effects of French maritime PBE (Pycnogenol^®^), with an emphasis on the research on PBE's metabolic kinetics and its potential health benefits. The polyphenols found in PBE have been linked to improve inflammation, gastrointestinal disturbances, and ADHD, as well as to modulate gut microbiota. While further research is needed to fully understand the long-term benefits and mechanisms, PBE represents a compelling addition to a balanced nutritional regimen, offering a natural avenue to promote a healthy wellbeing. Following aspects are provided as references for further research in the coming future.

1. Isolation and purification of nutraceutical components in PBE.
2. Identification of metabolites, including intermediate and end products.
3. Nutritional Metabolic-Kinetics of PBE as food preservatives, including both nutritional function and food quality, identification of target organelles or molecules.
4. Human randomized controlled trials (RCTs) of the nutritional effects of PBE.
